# Commentary: Retinal Waves Modulate an Intraretinal Circuit of Intrinsically Photosensitive Retinal Ganglion Cells

**DOI:** 10.3389/fncir.2017.00113

**Published:** 2018-01-08

**Authors:** Yu-Chieh D. Chen

**Affiliations:** Interdepartmental Neuroscience Program, University of California, Riverside, Riverside, CA, United States

**Keywords:** retinal waves, ipRGCs, gap junction, developing retina

In vertebrates, patterned spontaneous activity is found in many developing neuronal networks, including the retina, spinal cord, cortex, hippocampus, cochlea, and cerebellum. Both electrical and chemical synaptic transmission contribute to the propagation of patterned spontaneous activity, which is essential for refining immature neuronal circuits (Blankenship and Feller, 2010). Electrical synaptic transmission relies on gap junctions for rapid intercellular communication and signal integration. Gap junctions play an important role in synchronizing neuronal activity, thus performing diverse functions within the central nervous system, which is particularly evident in the vertebrate retina. For example, these gap junction coupling networks exist among different retinal neuronal types, and the coupling strength is tightly regulated by ambient illumination, as well as circadian rhythms (Bloomfield and Volgyi, 2009). Moreover, during a developmental critical period, both electrical and chemical transmission mediate patterned spontaneous activity termed retinal waves, which propagate throughout the retinal ganglion cell (RGC) layer in a wave-like manner. Among three different stages of retinal waves, stage II cholinergic waves have been vigorously studied for their underlying mechanisms and roles in visual system development, such as eye-specific segregation and retinotopic refinement of retinofugal projections (Huberman et al., 2008).

A remarkable feature of retinal waves is their robust recovery following perturbation. For example, the recovered gap junction waves are still observed in mice with blockage of stage II cholinergic waves either by pharmacological inhibition with the nicotinic acetylcholine receptor (nAChR) antagonist, dihydro-β-erythroidine (DHβE), or by genetic elimination of the β2 subunit of the nAChR (Stacy et al., 2005; Sun et al., 2008; Stafford et al., 2009; Kirkby and Feller, 2013). Such homeostatic responses ensure the persistence of patterned spontaneous activity for proper neuronal circuit development. However, the mechanism underlying wave recovery remains unclear. Recently, one study suggested that gap junction coupling of intrinsically photosensitive retinal ganglion cells (ipRGCs) contributes to the underlying homeostatic transition from cholinergic waves to recovered gap junction waves (Kirkby and Feller, 2013). In addition, another study showed that light acts through ipRGCs to modulate cholinergic waves and eye-specific segregation of RGC axons in the dorsal lateral geniculate nucleus (Renna et al., 2011). This suggests that ipRGCs may regulate stage II cholinergic waves under normal circumstances, whereas disruption of cholinergic waves could modulate gap junction coupling of ipRGCs and reactivate the gap junction waves. Although evidence for this bidirectional regulation between retinal waves and ipRGCs is emerging, the mechanism underlying retinal wave modulation of ipRGCs, and whether ipRGCs contribute to early non-image-forming light sensitivity in the developing retina, remains to be established. With a combination of calcium imaging, tracer coupling and electrophysiological recordings, a recent article in *The Journal of Neuroscience* by Arroyo et al. (2016) demonstrated that stage II cholinergic waves modulate dopaminergic signaling, which regulates the extent of ipRGC gap junction coupling, thereby contributing to early light responses in the developing retina.

To understand the effects of waves on light responses in the developing retina, Arroyo et al. (2016) examined the number of light-responsive cells upon blocking stage II cholinergic waves via DHβE treatment. They found that the number of cells with light-evoked calcium transients increased two-fold under the blockade of stage II cholinergic waves, and this effect persisted following additional application of synaptic blockers for GABAergic, glutamatergic, and glycinergic inputs. This result suggested that gap junction coupling, but not chemical synaptic transmission, contributes to the increase in the number of light-responsive cells (Arroyo et al., 2016). Since ipRGCs are the sole photoreceptors to transmit light response during the early postnatal stage, the authors next examined whether ipRGC gap junction networks are modulated by retinal waves. The authors filled single GFP-expressing ipRGCs from *Opn4-EGFP* mice with the gap junction-permeable tracer neurobiotin, and quantified cells expressing either marker upon wave blockade. Interestingly, blocking cholinergic waves increased the number of neurobiotin labeled cells, suggesting that the extent of ipRGC coupling to other neurons is regulated by the cholinergic waves. Additionally, the number of cells labeled by both neurobiotin and GFP increased slightly but significantly upon wave blockade, suggesting that the ipRGCs form gap junction networks with other ipRGCs, and the degree of coupling increases upon wave blockade (Arroyo et al., 2016). To further identify the cells types that are coupled with ipRGCs, co-labeling experiments with various cell-type specific markers showed a high percentage of neurobiotin-labeled cells were Brn3b^+^/Brn3a^−^, a hallmark characteristic for ipRGCs, but not conventional RGCs (Arroyo et al., 2016), supporting that ipRGCs form these networks among themselves.

Although these results suggest that ipRGCs indeed anatomically couple with one another, the authors did not point out the fact that most of the tracer-coupled cells are not positive for melanopsin (a marker for ipRGCs) in control or wave blocked retinas. For example, the authors described that around two-thirds of neurobiotin-positive cells in the control tracer coupling experiment were not labeled by *Opn4-EGFP*, suggesting that a low percentage of these cells were melanopsin-positive ipRGCs (Arroyo et al., 2016). The subsequent co-labeling experiments also failed to reveal the identity of these cell types in the ipRGCs gap junction networks. This might result from the fact that melanopsin expression level decreases during early retinal development (Sekaran et al., 2005; Sexton et al., 2015), and that some subtypes of ipRGCs were melanopsin immunonegative, which would make melanopsin immunosignal an unsuitable marker for ipRGCs. Alternatively, although ipRGCs mainly extend their dendrites into the inner plexiform layer of the inner retina, where they may make contact with amacrine cells and other RGCs, some of their dendrites are shown to extend further to the outer plexiform layer of the outer retina in the early postnatal stage (Renna et al., 2015). This raises the possibility that the non-ipRGC tracer-coupled cells are located outside the inner retina. Future co-labeling experiments with extensive markers covering more cell types throughout the retina in both control and wave blocked retinas will yield deeper insights into the identity of ipRGC-coupled cells.

Next, Arroyo et al. (2016) uncovered dopaminergic signaling as the underlying mechanism for wave-dependent modulation of ipRGC gap junction networks. They used the novel cell-based neurotransmitter fluorescent engineered reporters (CNiFERs), which are engineered cells expressing the dopamine receptor D2 (DRD2) and a FRET-based Ca^2+^ indicator. The CNiFERs show an enhanced FRET ratio when there is an increase in extracellular dopamine. When depositing CNiFERs on the inner limiting membrane of the retina and simultaneously performing whole-cell voltage clamp and FRET imaging, the authors found a correlation between retinal waves and FRET transients, strongly suggesting that retinal waves induce dopamine release (Arroyo et al., 2016). Subsequent pharmacological blockade of DRD1, but not DRD2 increased the number of light-responsive cells, likely through increasing the extent of ipRGC coupling networks. These results suggest that wave-dependent modulation of ipRGC gap junction networks is achieved through regulation of the strength of dopaminergic signaling in the developing retina. Intriguingly, one recent study in adult retina shows that ipRGCs signal to dopaminergic amacrine cells through intra-retinal axon collaterals, suggesting a possible reciprocal regulation in which ipRGCs control dopamine release, thereby modulating their gap junction networks (Prigge et al., 2016).

However, it is important to note that dopamine is released by dopaminergic amacrine cells and exerts its effect through volume transmission, which essentially affects all the retinal cell types expressing dopamine receptors. Among five dopamine receptor subtypes (DRD1-5), DRD1, 2, 4, and 5 are shown to be expressed in rodent ipRGCs (Sekaran et al., 2005; Van Hook et al., 2012), and DRD4 signaling is involved in circadian photoentrainment (Jackson et al., 2011). Future analysis of the role of these dopamine receptor subtypes in regulating ipRGCs gap junction networks should be conducted using both pharmacological and genetic manipulation of these receptors. Interestingly, activation of A1 and muscarinic acetylcholine receptors on ipRGCs by adenosine and acetylcholine, respectively, has recently been shown in neonatal retina to influence light responses (Sodhi and Hartwick, 2014, 2016). The way in which other neurotransmission pathways interact to modulate the extent of ipRGC gap junction networks might be more complex than previously imagined.

In conclusion, Arroyo et al. (2016) provides mechanistic insights into how changes in dopaminergic signaling modulate the extent of ipRGC gap junction networks in the absence of cholinergic waves in the developing retina. In the presence of cholinergic waves, ipRGC gap junction networks contribute to the early non-image-forming light sensitivity, suggesting that there is a homeostatic activity-dependent regulation of ipRGC gap junction networks in the developing retina. Their work presents a beautiful example of bidirectional regulation of ipRGC gap junction networks and spontaneous activity in early neuronal circuit development. In fact, previous studies show that sister neurons coupled via gap junctions in early development tend to form excitatory synapses with each other in the visual cortex (Li et al., 2012; Yu et al., 2012). Together, the activity-dependent regulation of gap junction coupling networks might be a conserved feature in the developing nervous systems.

## Author contributions

The author confirms being the sole contributor of this work and approved it for publication.

### Conflict of interest statement

The author declares that the research was conducted in the absence of any commercial or financial relationships that could be construed as a potential conflict of interest.
